# Can financial retention incentives slow health worker migration? PPP-adjusted salary differentials in Zimbabwe’s donor-dependent health system

**DOI:** 10.1186/s12960-026-01074-y

**Published:** 2026-05-08

**Authors:** Gavin George

**Affiliations:** 1 Health Economics and HIV and AIDS Research Division (HEARD), University of KwaZulu-Natal, Durban, South Africa; 2 Division of Social Medicine and Global Health, Lund University, Lund, Sweden

**Keywords:** Health worker migration, Purchasing power parity, Salary differentials, Retention incentives, Donor-dependent health systems

## Abstract

**Background:**

Large-scale migration of health workers from low-income countries continues to undermine health system capacity, particularly in contexts characterised by fiscal constraint and donor dependence. While retention incentives are widely used to mitigate attrition, their ability to offset international wage differentials remains contested. Zimbabwe provides a salient case, having implemented a long-standing Health Worker Retention Scheme (HWRS), largely financed by external partners, to stabilise critical cadres within the public health sector. This study aimed to quantify PPP-adjusted international salary differentials for selected Zimbabwean health worker cadres and to assess the extent to which donor-financed retention incentives narrow these gaps.

**Methods:**

This was a cross-country comparison of annual remuneration for three cadres: professional nurses, medical officers, and medical specialists, using a purchasing power parity (PPP) framework. Salaries in Zimbabwe, South Africa, the United Kingdom, and the United States were compiled from official public-sector pay schedules and adjusted using International Comparison Program (ICP) 2021 PPP conversion factors. Zimbabwean salaries were analysed under three scenarios: base salary; base salary plus USD-denominated Government of Zimbabwe (GOZ) allowances; and total remuneration including GOZ allowances and Global Fund-financed HWRS top-ups. PPP-adjusted salary gaps were calculated relative to Zimbabwean health workers’ base salary.

**Results:**

Combined USD-denominated Government of Zimbabwe allowances and Global Fund-financed HWRS top-ups substantially increased PPP-adjusted salaries across all cadres, more than doubling remuneration for medical officers and specialists and nearly tripling earnings for nurses. Despite these increases, PPP-adjusted salaries in South Africa, the United Kingdom, and the United States remained several multiples higher than those in Zimbabwe.

**Conclusion:**

Combined domestic and donor-financed retention incentives meaningfully improve Zimbabwe’s relative salary position but do not fundamentally alter the international remuneration gradient that underpins health worker migration. In donor-dependent health systems, such schemes function as stabilising mechanisms rather than long-term solutions. Sustainable retention will require complementary strategies addressing career progression, working conditions, and the global governance of health worker mobility.

## Introduction

Health systems depend fundamentally on the availability, motivation, and adequate remuneration of health workers. Yet the global health labour market remains characterised by deep and persistent imbalances. Recent estimates project a global shortfall of approximately 10–11 million health workers by 2030, with the burden concentrated overwhelmingly in low- and lower-middle-income countries (LMICs) [3, 36]. These shortages coexist with growing demand in high-income countries (HICs), ageing populations, and increasingly institutionalised international recruitment pipelines, creating powerful structural pull factors which facilitate health worker migration [25].

High-income destination countries have become increasingly reliant on internationally trained health professionals to strengthen their health systems. In the United Kingdom, for example, nearly half of hospital doctors are now internationally trained, reflecting sustained dependence on overseas recruitment to fill domestic workforce gaps [9, 10]. Similar patterns are evident in other major destination countries, raising long-standing concerns about the implications of global health worker mobility for source-country health systems [37, 38]. In response, the WHO Global Code of Practice on the International Recruitment of Health Personnel provides a normative framework for ethical recruitment, emphasising the need to balance individual mobility rights with health system sustainability and to promote shared responsibility between source and destination countries [35]. However, recent assessments indicate that while the Code remains relevant, international recruitment has become increasingly institutionalised and demand-driven, with destination countries continuing to benefit from the migration of foreign-trained health workers without commensurate investment in source-country health systems [39].

A substantial body of literature identifies remuneration differentials as one of the most salient drivers of health worker migration, operating alongside working conditions, career progression, professional recognition, and broader socio-economic security [12, 29]. For source countries facing macroeconomic instability, wage erosion, and constrained fiscal space, financial incentives are often decisive. In Zimbabwe, concerns about outward mobility and its system-wide effects have been documented over more than two decades, with recent analyses highlighting persistent salary compression, exchange-rate volatility, and limited domestic capacity to compete with regional and global labour markets [22].

Analytically, assessing the true financial incentive to migrate requires moving beyond nominal salary comparisons. Differences in price levels across countries mean that nominal wages alone provide a misleading picture of relative welfare. Purchasing power parity (PPP) adjustment offers a standardised approach by converting national-currency salaries into international dollars that reflect equivalent real consumption potential [33]. This approach has been widely used in empirical analyses of health worker migration incentives, including prior work examining salary gaps between LMICs and major destination countries [11, 13, 14].

Zimbabwe provides a particularly salient case of structurally mediated health workforce vulnerability. The 2022 Health Labour Market Analysis (HLMA) estimated approximately 74,000 trained health workers across 72 cadres, yet only around two-thirds were actively participating in the labour market, this despite public-sector vacancy rates approaching 17% [21, 34]. Applications for Certificates of Good Standing, commonly used as a proxy for emigration intent, had risen sharply among nurses and doctors, reflecting sustained external pull factors and domestic push pressures [21]. These dynamics unfold in a context of pronounced donor dependence, with external financing underpinning large shares of Zimbabwe’s health wage bill and priority programmes [34, 40].

In response to recurrent waves of attrition, Zimbabwe had implemented a series of health worker retention interventions since the mid-2000s, culminating in the establishment and evolution of the Health Worker Retention Scheme (HWRS) [21]. Prior analyses of Zimbabwe’s labour market documented the impact of the economic crises and structural constraints on health worker employment and motivation, which stimulated early retention strategies covering financial and non-financial incentives [4, 21]. The HWRS was introduced as a structured retention allowance mechanism in this broader policy environment and has since undergone multiple design iterations and funding modalities, with a strong emphasis on stabilising cadres facing the highest attrition risk [21]. Supported by development partners, most prominently the Global Fund, this scheme has functioned as a targeted salary top-up mechanism aimed at improving public-sector health worker retention amid limited fiscal space and continuing outward mobility pressures [21]. The specific funding cycle evaluated in this paper corresponds to the 2021–2023 Global Fund New Funding Mechanism (NFM) 3 period, during which the HWRS was refined to prioritise critical cadres and locations experiencing elevated attrition risk [21].

Evidence from earlier programme evaluations of the HWRS suggested that financial retention incentives had contributed to short-term reductions in attrition, particularly among targeted cadres and in high-vacancy settings [8, 21]. These assessments reported improvements in retention and reductions in vacancy rates following the introduction of allowance top-ups, although effects varied across cadres and were sensitive to broader macroeconomic conditions [8]. More recent labour market analyses indicated that while attrition pressures remained high, targeted financial incentives had been associated with relative stabilisation of selected cadres, particularly medical officers and specialists, compared to preceding periods [21]. Nevertheless, even with retention allowances, average earnings for many health workers remained below estimated reservation and transfer wages, indicating that substantial economic incentives to migrate persisted. These pressures are especially acute for skilled cadres such as medical officers, specialists, and professional nurses, whose international labour market opportunities are most pronounced [20, 21]. This pattern is consistent with literature which suggests that while retention incentives may mitigate short-term attrition, they operate within a broader structural context in which international wage differentials and labour market opportunities continue to exert strong influence on mobility decisions [22, 29].

Against this backdrop, understanding whether retention schemes meaningfully narrow the real income gap between Zimbabwe and destination countries is critical for policy design. Without situating national salary top-ups within an internationally comparable PPP-adjusted framework, assessments of effectiveness risk overstating the protective effect of donor-supported interventions or understating the continued pull of external labour markets.

Building on established PPP-based salary comparison methodologies, this paper quantifies PPP-adjusted remuneration gaps between Zimbabwean health workers, specifically focusing on professional nurses, medical officers, and medical specialists against their counterparts in South Africa, the United Kingdom, and the United States, using International Comparison Program (ICP) 2021 PPPs. This paper specifically examines the extent to which the HWRS during the 2021–2023 funding cycle narrowed these gaps, thereby informing more realistic and fiscally grounded approaches to health workforce retention in aid-dependent and economically constrained settings.

## Methods

### Study design

A cross-country comparative analysis of health worker remuneration was conducted using a purchasing power parity (PPP) framework to estimate differences in real earnings across selected countries. The analysis focused on three cadres that are both critical to Zimbabwe’s health system and highly mobile internationally: professional nurses (registered nurses), medical officers, and medical specialists. The study design mirrors established PPP-adjusted salary comparison approaches used in prior analyses of health worker migration [11, 13, 14], while updating data sources and explicitly incorporating retention allowances implemented under Zimbabwe’s HWRS. Remuneration estimates were standardised to reflect urban-aligned or central public-sector salary levels across all countries, recognising that within-country geographic variation exists but is not explicitly modelled.

### Data sources and comparator countries

Zimbabwean remuneration data were compiled from official government pay schedules and publicly available evaluations of the HWRS covering the 2021–2023 Global Fund funding cycle, with contextual information on workforce composition, vacancy rates, and attrition trends drawn from the Zimbabwe Comprehensive Health Labour Market Analysis (HLMA) [21].

Comparator country salaries were obtained from authoritative public sources for major destination countries commonly identified in the Zimbabwean migration literature [21]. South African comparator salaries were drawn from the National Department of Public Service and Administration Occupation-Specific Dispensation (OSD) salary scales for 2023/2024 [7]. United Kingdom salaries were sourced from National Health Service (NHS) pay schedules, and published guidance on NHS doctor pay [2, 24]. United States salaries were obtained from the U.S. Bureau of Labor Statistics (BLS) Occupational Outlook Handbook for physicians and registered nurses [30, 31] (BLS 2023).

Where salary scales were presented as ranges or bands, mid-point values were selected to ensure consistency and comparability across countries and cadres, in line with prior PPP-based studies [13, 14]. Across all countries, remuneration estimates were standardised to reflect central or typical public-sector salary levels rather than location-specific variation.

### Salary compilation and exchange-rate conversion

All salaries were compiled on an annual basis and initially recorded in national currency units (NCU). For descriptive purposes only, salaries were converted to United States dollars (USD) using average market exchange rates corresponding to the PPP reference period, drawing on International Monetary Fund International Financial Statistics [18].

In the Zimbabwean context, remuneration is characterised by a hybrid structure comprising local currency (Zimbabwean dollar, ZWL) salary components and USD-denominated allowances. Base government salaries and selected domestic allowances are primarily paid in ZWL, while additional allowances, including fixed Government of Zimbabwe (GOZ) USD payments and Global Fund-financed HWRS top-ups, are denominated and disbursed in USD or USD-indexed equivalents. HWRS payments are administered through externally supported mechanisms and disbursed outside standard government payroll systems [16, 21].

Given exchange-rate volatility and the presence of parallel market dynamics, ZWL-denominated components were converted to USD using market-aligned exchange rates to better reflect the effective purchasing environment faced by health workers, consistent with prior PPP-based analyses [11, 13, 14]. USD-denominated components were retained at face value.

For analytical consistency, total remuneration was constructed by aggregating (i) ZWL-denominated government salary components, (ii) fixed GOZ USD allowances, and (iii) cadre-specific HWRS top-ups prior to PPP adjustment. Market-exchange-rate USD values are reported for transparency but are not used as the primary analytic metric. The structure of Zimbabwean remuneration and incentive levels is summarised in Table 1.

**Table 1:** Structure of Zimbabwean health worker remuneration and USD-denominated incentives (monthly values)

Cadre	Government salary (ZWL, monthly)	Government salary (USD, monthly)*	GOZ USD allowance (monthly)	HWRS (GF) top-up (monthly)	Total monthly package (USD)
Specialist/consultant	1,414,872	457	175	350	982
Medical officer	1,371,528	268	175	300	743
Professional nurse (urban, representative category)**	953,568	145	175	90	410

(a) Government salary reflects ZWL-denominated remuneration converted to USD using market-aligned exchange rates, (b) Nurse category reflects a representative urban-aligned mid-level nurse grade; remuneration varies across nursing categories and locations, (c) GOZ allowance comprises USD 100 (standard allowance) and USD 75 (COVID-related allowance), (d) HWRS top-ups are financed through the Global Fund and vary by cadre

*Government salary (USD, monthly) refl ects conversion of ZWL-denominated base salary using market-aligned exchange rates and ispresented for descriptive purposes only

**The nurse category refl ects a representative urban-aligned mid-level nurse grade; remuneration varies across nursing cadres andgeographic locations

### Purchasing power parity adjustment

To enable meaningful cross-country comparison of real earnings, salaries were adjusted using PPP conversion factors from the World Bank ICP 2021, which provides internationally comparable estimates of relative price levels and purchasing power [33]. PPP conversion factors express the number of national currency units required to purchase the same basket of goods and services as one international dollar, thereby equalising purchasing power across countries [26, 27].

PPP-adjusted salaries were calculated as

$${\text{PPP - adjusted}}\,{\mathrm{salary}}\,({\mathrm{Int}}\$ ) = \frac{{{\mathrm{Salary}}\,({\mathrm{NCU}})}}{{{\mathrm{PPP}}\,{\mathrm{conversion}}\,{\mathrm{factor}}\,({\mathrm{NCU}}\,{\mathrm{per}}\,{\mathrm{Int}}\$ )}}.$$

By definition, the United States was assigned a PPP conversion factor of 1.0, such that U.S. salaries in USD are numerically equal to international dollars. PPP-adjusted salaries constitute the primary outcome measure for all cross-country comparisons, as they reflect relative spending power rather than nominal exchange-rate differences [13, 14].

### Integration of the HWRS

To assess the extent to which retention incentives alter Zimbabwe’s relative competitiveness, three remuneration scenarios were constructed for each cadre:

1. Base salary (ZWL-denominated public-sector remuneration)
2. Base salary plus USD-denominated GOZ allowances
3. Total remuneration (base salary plus GOZ allowances and Global Fund-financed HWRS top-ups).

Allowance values were added to base salaries prior to PPP adjustment. This approach enables comparison of Zimbabwe’s position both with and without donor-supported retention incentives, consistent with prior analyses of salary adjustments and their impact on PPP outcomes [11].

### Outcome measures and comparative calculations

For each cadre and country, we report:

- annual salary in national currency units (NCU),
- USD-equivalent salary based on market exchange rates (for descriptive purposes),
- PPP-adjusted salary expressed in international dollars, and
- percentage differences relative to Zimbabwe’s base salary PPP-adjusted earnings.

Percentage gaps were calculated as

$${{\% Gap}} = \frac{{{\mathrm{Comparator}}\,{\mathrm{PPP}}\,{\mathrm{salary}} - {\mathrm{Zimbabwe}}\,{\mathrm{PPP}}\,{\text{salary }}}}{{{\mathrm{Zimbabwe}}\,{\mathrm{PPP}}\,{\mathrm{salary}}}} \times 100.$$

Percentage gaps were first calculated relative to Zimbabwe’s base salary PPP-adjusted earnings. Additional percentage gaps were computed using Zimbabwe’s total remuneration (base salary plus USD-denominated GOZ allowances and Global Fund-financed HWRS top-ups) to illustrate the extent to which combined retention incentives narrow, but do not eliminate, international salary differentials.

### Sensitivity checks and limitations

The analysis prioritised ICP-2021 PPPs as the most recent globally comparable benchmark [33]. Sensitivity checks examined the use of adjacent-year exchange rates for USD conversion and alternative notch selections within UK NHS and South African OSD pay scales, these did not materially alter the direction or magnitude of observed gaps. The analysis is limited to public-sector salary scales and does not capture private-sector earnings, non-wage benefits, or informal income sources in destination countries. Given Zimbabwe’s macroeconomic volatility, absolute salary levels may fluctuate over time; however, PPP-adjusted ratios are comparatively stable and form the basis of interpretation.

## Results

Tables 2–4 present annual base salaries in national currency units, market-exchange-rate US dollar equivalents, PPP-adjusted salaries expressed in international dollars, and percentage gaps relative to Zimbabwean health workers. Zimbabwean remuneration is shown under three scenarios: Base salary only, Base salary plus USD-denominated GOZ allowances, and Base salary plus GOZ allowances and Global Fund-financed HWRS top-ups. These scenarios illustrate the extent to which combined domestic and donor-financed retention incentives modify Zimbabwe’s relative position in international salary comparisons. Across all cadres, PPP-adjusted salaries in South Africa, the United Kingdom, and the United States substantially exceeded Zimbabwean salaries under the base salary scenario. The inclusion of GOZ allowances and HWRS top-ups resulted in substantial increases in Zimbabwean earnings and narrowed international differentials, but did not eliminate them.

**Table 2:** Annual remuneration and PPP-adjusted salary comparisons for Specialists

Country	Cadre	Scenario	Base salary (NCU, annual)	NCU	USD (market, annual)	PPP-adjusted USD (Intl$, annual)	% gap vs. Zim (Base salary only)	% gap vs. Zim (Total remuneration)
Zimbabwe	Specialist (F)	Base salary only	1,414,872	ZWL	5484	9793	0.0	−53.5
Zimbabwe	Specialist (F)	Base salary + GOZ allowance	1,414,872	ZWL	7584	13,543	38.3	−35.6
Zimbabwe	Specialist (F)	Base salary + GOZ allowance + GF	1,414,872	ZWL	11,784	21,043	114.9	0.0
South Africa	Specialist	OSD mid-range	1,427,352	ZAR	77,363	193,408	1875.0	819.1
United Kingdom	Specialist	NHS mid-point	112,356	GBP	140,445	165,229	1587.2	685.2
United States	Specialist	BLS OEWS mean	239,200	USD	239,200	239,200	2342.6	1036.7

(a) NCU values reflect ZWL-denominated base salary only, (b) USD values for Zimbabwe include; Base salary (converted from ZWL using market-aligned exchange rates), USD 175/month GOZ allowance (USD 2100 annually), and USD 350/month Global Fund (HWRS) top-up (USD 4200 annually), (c) PPP-adjusted values are expressed in international dollars (US = 1.00) using ICP 2021 conversion factors, (d) Percentage gaps are calculated relative to Zimbabwe under; (i) Base salary only scenario, and (ii) total remuneration (Base salary + GOZ allowances + Global Fund)

As shown in Table 2, Zimbabwean medical specialists earned a PPP-adjusted annual salary of Int$9793 under the base salary scenario. With the inclusion of USD-denominated GOZ allowances, this increased to Int$13,543, representing a 38.3% increase relative to the base salary baseline. When both GOZ allowances and Global Fund-financed HWRS top-ups were incorporated, PPP-adjusted annual remuneration increased further to Int$21,043, corresponding to a 114.9% increase relative to base salary.

Despite these substantial increases, large international differentials remained. PPP-adjusted salaries for specialists were Int$193,408 in South Africa, Int$165,229 in the United Kingdom, and Int$239,200 in the United States. Relative to Zimbabwe’s base salary PPP-adjusted earnings, these represent gaps of 1875, 1587, and 2343%, respectively. When Zimbabwe’s total remuneration (base salary plus GOZ allowances and Global Fund-financed HWRS top-ups) is used as the comparator, the gaps narrow considerably but remain pronounced, at 819% for South Africa, 685% for the United Kingdom, and 1037% for the United States.

Table 3 shows a similar pattern for medical officers, although the relative gains from USD-denominated incentives are more pronounced. Zimbabwean medical officers earned a PPP-adjusted annual salary of Int$9493 under the base salary scenario. With the inclusion of USD-denominated Government of Zimbabwe (GOZ) allowances, this increased to Int$13,243, representing a 39.5% increase relative to the base salary baseline. When both GOZ allowances and Global Fund-financed HWRS top-ups were incorporated, PPP-adjusted annual remuneration increased further to Int$19,671, corresponding to a 107.2% increase relative to base salary.

**Table 3:** Annual remuneration and PPP-adjusted salary comparisons for medical officers

Country	Cadre	Scenario	Base salary (NCU, annual)	NCU	USD (market, annual)	PPP-adjusted USD (Intl$, annual)	% gap vs. Zim (Base salary only)	% gap vs. Zim (Total remuneration)
Zimbabwe	Medical Officer (E5)	Base salary only	1,371,528	ZWL	5316	9493	0.0	−40.4
Zimbabwe	Medical Officer (E5)	Base salary + GOZ allowance	1,371,528	ZWL	7,416	13,243	39.5	−16.8
Zimbabwe	Medical Officer (E5)	Base salary + GOZ allowance + GF	1,371,528	ZWL	11,016	19,671	107.2	0.0
South Africa	Medical Officer	OSD mid-range	1,080,681	ZAR	58,573	146,434	1442.5	644.3
United Kingdom	Medical Officer	NHS mid-point	50,701	GBP	63,376	74,560	685.4	279.0
United States	Medical Officer	BLS OEWS mean	239,200	USD	239,200	239,200	2419.8	1116.0

(a) NCU values reflect ZWL-denominated base salary only, (b) USD values for Zimbabwe include; Base salary (converted from ZWL), USD 175/month GOZ allowance (USD 2100 annually), and USD 300/month Global Fund (HWRS) top-up (USD 3600 annually), (c) PPP-adjusted values are expressed in international dollars (US = 1.00), (d) Percentage gaps are calculated relative to: (i) Base salary only scenario, and (ii) total remuneration (Base salary + GOZ allowances + Global Fund)

Comparator PPP-adjusted salaries remained substantially higher, at Int$146,434 in South Africa, Int$74,560 in the United Kingdom, and Int$239,200 in the United States. Relative to Zimbabwe’s base salary PPP-adjusted earnings, these correspond to percentage gaps of 1443, 685, and 2420%, respectively. When Zimbabwe’s total remuneration is used as the comparator, the gaps narrow considerably but remain substantial, at 644% for South Africa, 279% for the United Kingdom, and 1116% for the United States.

PPP-adjusted salary comparisons for nurses (Table 4) indicate a similar overall pattern, although starting from a substantially lower base. Zimbabwean nurses earned a PPP-adjusted annual salary of Int$3107 under the base salary scenario. With the inclusion of USD-denominated GOZ allowances, this increased to Int$6857, representing a 120.6% increase relative to the base salary. When Global Fund-financed HWRS top-ups were incorporated, PPP-adjusted remuneration increased further to Int$8785, corresponding to a 182.8% increase relative to the base salary baseline.

**Table 4:** Annual remuneration and PPP-adjusted salary comparisons for Nurses (urban-aligned category)

Country	Cadre	Scenario	Base salary (NCU, annual)	NCU	USD (market, annual)	PPP-adjusted USD (Intl$, annual)	% gap vs. Zim (Base salary only)	% gap vs. Zim (Total remuneration)
Zimbabwe	Nurse (urban, representative)	Base salary only	941,184	ZWL	1740	3107	0.0	−64.6
Zimbabwe	Nurse (urban, representative)	Base salary + GOZ allowance	941,184	ZWL	3840	6857	120.6	−21.9
Zimbabwe	Nurse (urban, representative)	Base salary + GOZ allowance + GF	941,184	ZWL	4920	8785	182.8	0.0
South Africa	Nurse	OSD mid-range	386,778	ZAR	20,964	52,409	704.6	496.6
United Kingdom	Nurse	NHS mid-point	30,639	GBP	38,299	45,057	591.7	412.9
United States	Nurse	BLS OEWS mean	93,600	USD	93,600	93,600	1336.9	965.7

(a) NCU values reflect ZWL-denominated base salary only, (b) USD values for Zimbabwe include; Base salary (converted from ZWL), USD 175/month GOZ allowance (USD 2100 annually), and USD 90/month Global Fund (HWRS) top-up (USD 1080 annually), (c) PPP-adjusted values are expressed in international dollars (US = 1.00), (d) The Zimbabwe nurse category reflects a representative urban-aligned mid-level nurse grade; remuneration varies across nursing cadres and locations, (e) Percentage gaps are calculated relative to; (i) Base salary only scenario, and (ii) total remuneration (Base salary + GOZ allowances + Global Fund)

In contrast, PPP-adjusted salaries were Int$52,409 in South Africa, Int$45,057 in the United Kingdom, and Int$93,600 in the United States. Relative to Zimbabwe’s base salary PPP-adjusted earnings, these correspond to percentage gaps of 705, 592, and 1337%, respectively. When Zimbabwe’s total remuneration is used as the comparator, the gaps narrow substantially but remain pronounced, at 497% for South Africa, 413% for the United Kingdom, and 966% for the United States.

Across all cadres examined, the inclusion of USD-denominated Government of Zimbabwe allowances and Global Fund-financed HWRS top-ups resulted in substantial increases in PPP-adjusted Zimbabwean salaries. Proportional gains were largest for nurses, reflecting their lower baseline remuneration, and remained substantial for both medical officers and specialists. Despite these increases, PPP-adjusted earnings in South Africa, the United Kingdom, and the United States remained several multiples higher than those in Zimbabwe across all cadres. These findings indicate that while combined domestic and donor-financed retention incentives meaningfully narrow salary differentials, they do not fundamentally alter the international remuneration gradient that underpins health worker migration.

## Discussion

This study applied a PPP framework to examine international salary differentials faced by selected Zimbabwean health worker cadres and to assess the extent to which combined domestic and donor-financed retention incentives modify these gaps. Consistent with prior PPP-based analyses of health worker migration, the findings confirm that large real income differentials persist between source and major destination countries, even after adjusting for differences in cost of living [11, 13, 14]. However, the present analysis extends this literature by explicitly modelling the combined effect of USD-denominated domestic allowances and donor-financed top-ups within a low-income, fiscally constrained setting. The results demonstrate that while such incentives can substantially increase remuneration and improve relative earnings, they are insufficient to offset the structural international wage differentials that underpin health worker migration.

While previous evaluations of the HWRS have documented reductions in attrition and improvements in short-term workforce stability [8, 21], these analyses have largely been confined to observed retention outcomes within Zimbabwe and have not systematically examined the scheme’s effect relative to international labour market conditions. The PPP-based analysis presented here does not measure migration behaviour directly, nor does it attempt to estimate the causal effect of remuneration on migration decisions. Rather, it provides a complementary lens by quantifying the extent to which HWRS allowances alter the relative financial attractiveness of remaining in the domestic health system compared to migrating.

From this perspective, although the HWRS improves the real earnings position of Zimbabwean health workers, the underlying international differential in earning potential remains large after accounting for cost-of-living differences. The implication is not that migration decisions are determined solely by wage differentials, but that the economic incentive to migrate, understood here as the gap in real earning potential, remains structurally significant even in the presence of retention incentives. Accordingly, the PPP analysis refines, rather than replaces, prior impact assessments, situating observed retention effects within a broader global labour market context and suggests that the HWRS functions primarily as a mitigating intervention that slows attrition rather than one that fundamentally alters migration incentives. This distinction is important for policy, as it underscores the limits of donor-financed top-up schemes in addressing structural drivers of health worker mobility.

Consistent with this interpretation, PPP-adjusted salaries in South Africa, the United Kingdom, and the United States remain several multiples higher than Zimbabwean public-sector salaries across all cadres examined. Although this study does not assess individual migration decisions or attribute causality, the persistence of these differentials is salient in light of a broader literature that identifies remuneration as one of several important pull factors shaping health worker mobility, alongside working conditions, career progression, and broader socio-economic security [22, 29].

Importantly, the results help clarify why outward mobility from Zimbabwe has remained high despite repeated policy interventions aimed at retention [21]. As shown in prior qualitative and mixed-methods studies, health workers often interpret salary levels relative to international reference points rather than domestic baselines, especially in contexts of economic volatility and currency instability [6, 22]. PPP-adjusted comparisons therefore provide a more accurate lens through which to interpret the structural pull exerted by high-income destination countries.

### The role and limits of the Health Worker Retention Scheme

The HWRS represents a long-standing and evolving policy response to recurrent waves of attrition in Zimbabwe’s public health sector. Introduced initially in the post-crisis period following economic collapse and dollarisation, the scheme has undergone multiple design iterations, funding modalities, and scale adjustments over time, with increasing reliance on external financing, most prominently from the Global Fund [8, 21].

Our findings show that, during the 2021–2023 funding cycle, USD-denominated allowances, including HWRS top-ups, produced substantial increases in PPP-adjusted earnings, particularly for specialists and medical officers. However, the persistence of large PPP-adjusted gaps after allowances are applied underscores the structural limits of salary top-ups as a standalone retention strategy. This echoes findings from Zimbabwe’s Results-Based Financing (RBF) experience, where financial incentives improved motivation and job satisfaction but were insufficient to offset broader systemic constraints, including workload pressures, career stagnation, and resource shortages [17, 23]. In this sense, the HWRS appears to function more as a stabilising mechanism than as a tool capable of neutralising international migration incentives.

### Donor dependence and sustainability concerns

A critical contribution of this study is its explicit quantification of retention effects within a donor-dependent remuneration framework. The HWRS allowances examined here are largely financed through external resources, raising questions about sustainability in the context of shifting global aid priorities and heightened uncertainty in health financing. Previous analyses of Zimbabwe’s health labour market and financing context have highlighted the structural constraints associated with absorbing donor-funded incentives into the public-sector wage bill, particularly under conditions of limited fiscal space and macroeconomic instability [8, 21, 34]. More broadly, evidence from Zimbabwe suggests that external assistance has often functioned to support service delivery rather than to establish durable, domestically financed system capacity [19].

The PPP results extend this literature by providing a quantified estimate of the underlying remuneration gap in real purchasing power terms. The analysis demonstrates that the removal or reduction of donor-financed allowances would widen already substantial salary differentials relative to international labour markets. While this study does not model fiscal scenarios or predict behavioural responses, these findings underscore the scale of the adjustment that would be required to sustain current remuneration levels through domestic financing alone. In the absence of such adjustments, reductions in retention incentives may increase the relative economic attractiveness of migration [22]. From a policy perspective, this highlights the vulnerability of retention strategies that rely on externally financed top-ups, and the potential risks associated with abrupt withdrawal of such incentives for key health cadres, particularly specialists, medical officers, and experienced nurses.

### Implications for policy and ethical recruitment

The findings reinforce the need to situate salary-based retention interventions within a broader package of HRH strategies, consistent with international evidence that bundled approaches are more effective than isolated financial incentives [1, 17]. The PPP analysis contributes to this perspective by providing a benchmark for assessing the scale of financial adjustments required to influence retention. In practice, this suggests that modest or uniform salary adjustments are unlikely to substantially shift retention outcomes where large international wage differentials persist, and that more targeted and context-specific approaches may be required. While PPP-adjusted salary convergence with high-income countries is unlikely in the short to medium term, retention strategies may still yield gains by improving predictability, reducing income volatility, and signalling institutional commitment to the workforce.

Beyond confirming the existence of international wage differentials, the PPP framework provides a structured basis for assessing the feasibility and targeting of retention policies. This has several practical implications. First, it enables policymakers to assess the feasibility of retention strategies relative to the size of the underlying labour market imbalance, highlighting that full wage convergence with destination countries is fiscally unrealistic in the short to medium term. Second, it supports prioritisation by identifying cadres for whom relative differentials are largest and where targeted incentives may yield the greatest marginal retention benefit. Third, it allows for more realistic calibration of financial interventions by distinguishing between adjustments that improve income stability and those that would be required to materially reduce international earning differentials. Finally, the PPP framework provides a common metric through which domestic policy options can be assessed alongside international labour market conditions, facilitating more transparent dialogue between governments, donors, and global health actors.

At the international level, the magnitude of the PPP-adjusted salary differentials documented in this study have further important implications for debates on ethical recruitment, global labour markets, and responsibility for health system sustainability in source countries. As Zimbabwe continues to lose health workers to countries that have increasingly institutionalised international recruitment mechanisms, donor-financed retention schemes function as temporary stabilisation tools rather than durable solutions. This raises ethical and policy concerns about the extent to which destination countries benefit from investments made by resource-constrained health systems, while the costs of workforce reproduction and retention are borne domestically or by external donors.

While the WHO Global Code of Practice on the International Recruitment of Health Personnel provides an important normative framework for ethical recruitment, its voluntary nature and limited enforcement have constrained its effectiveness [41]. Emerging analyses suggest that current recruitment systems may operate in ways that are structurally asymmetrical, with high-income countries drawing on the health workforce capacity of lower-income settings without commensurate reinvestment [5, 15, 32].

Within this context, more substantive policy approaches warrant consideration, including bilateral agreements incorporating financial transfers or training support, co-financing of health workforce development, and mechanisms linking international recruitment to investment in source-country health systems. The PPP-adjusted gaps identified in this study provide a quantitative indication of the scale of these imbalances. By demonstrating that large real income differentials persist even after cost-of-living adjustment, the analysis strengthens the case for moving beyond voluntary ethical principles towards more operationalised frameworks of shared responsibility between source and destination countries. Without such mechanisms, the sustainability of donor-financed retention strategies in countries such as Zimbabwe will remain uncertain, and global health workforce distribution will continue to reflect entrenched structural inequalities.

### Limitations

This analysis has several limitations. First, the analysis uses urban-aligned remuneration levels for Zimbabwean nurses and does not explicitly model rural allowances or hardship incentives. However, salary variation by geography is a common feature across all comparator countries, including rural allowances in South Africa and regional pay variation in the United Kingdom and United States. As such, the analysis focuses on central salary benchmarks to ensure comparability, rather than attempting to capture within-country heterogeneity. Additionally, the focus is also on public-sector salary scales and does not capture private-sector earnings or non-wage benefits in destination countries. Following, while PPP adjustment improves comparability, it cannot fully account for differences in taxation, social protection, or long-term career earnings. Finally, this study does not estimate causal effects of the HWRS on migration outcomes; rather, it quantifies the extent to which retention incentives alter relative salary positions. Nonetheless, by situating Zimbabwe’s remuneration within an international PPP framework, the analysis provides a robust basis for policy discussion.

## Conclusion

In conclusion, this study shows that combined domestic and donor-financed retention incentives, comprising USD-denominated Government of Zimbabwe allowances and Global Fund-supported HWRS top-ups, substantially increase health worker remuneration but narrow, rather than eliminate the international salary differentials that underpin migration from Zimbabwe. PPP-adjusted comparisons highlight both the value and the structural limits of financial retention schemes in donor-dependent health systems.

From a policy perspective, these findings suggest that while targeted salary top-ups can play an important short-term stabilising role, they are unlikely to be sufficient as standalone interventions. More realistic and fiscally grounded retention strategies will require a combination of measures, including improved predictability and structure of remuneration, strengthened career progression pathways, and investments in working conditions that enhance job satisfaction and professional recognition. In parallel, greater attention is needed at the global level to the governance of health worker mobility, including more consistent adherence to ethical recruitment frameworks and consideration of mechanisms to support source countries facing sustained workforce losses.

## Data Availability

All data supporting the findings of this study are available in the public domain.
